# Congenital heart disease in children in Hawassa, Ethiopia: a multicenter study on patterns, complications, survival, and mortality predictors

**DOI:** 10.1186/s12872-026-06009-y

**Published:** 2026-05-26

**Authors:** Mohammed Nasir, Mekdes Wendmagegn, Getasew Ademu

**Affiliations:** Department of Pediatrics, Division of Cardiology, Hawassa University, Hawassa, 1560, Ethiopia

**Keywords:** congenital heart disease, pattern, complication, survival, mortality, predictors

## Abstract

**Background:**

Given the limited data from developing countries, this study aimed to examine the patterns of congenital heart disease, associated complications, management, survival and mortality rates, and independent predictors of mortality in children.

**Methodology:**

This retrospective multicenter follow-up study was conducted at five hospitals in Hawassa, Ethiopia, from April 1 to July 1, 2025, including children seen between January 1, 2015, and January 1, 2025. Patterns of congenital heart disease, complications, and management were summarized as frequencies and percentages. Mortality incidence was calculated per 1,000 person-years. Kaplan–Meier analysis estimated survival, and Cox proportional hazards regression identified independent mortality predictors.

**Results:**

A total of 1,251 children met the inclusion criteria. Acyanotic congenital heart diseases predominated (1,110; 88.7%), with Ventricular septal defect (384; 30.7%), patent ductus arteriosus (207; 16.5%), and secundum atrial septal defect (158; 12.6%) being the most common. Over a median follow-up of 5 years (IQR: 4–7), 1,043 children (83.4%) developed at least one complication, most frequently New York Heart Association (NYHA)/Modified Ross class III/IV heart failure (HF) (204; 16.3%) and isolated pulmonary hypertension (PH) (168; 13.4%). Most children were managed medically (1,210; 96.7%), 23 (1.8%) received follow-up care only, and 18 (1.4%) underwent intervention (17 surgeries and 1 catheter-based procedure). Among the 1,013 children (81.0%) with indications for surgical or catheter-based intervention, only 18 (1.4%) actually received the procedure, indicating that the vast majority of eligible children did not undergo intervention. Overall survival was 96.6%, 95.0%, 90.2%, and 84.6% at 1, 3, 5, and 10 years, with a mortality incidence of 18.9 per 1,000 person-years (95% CI: 15.9–22.4). Mortality risk was higher in children with syndromic association (HR = 2.9; 95% CI: 2.0–4.3; *p* = 0.02), severe acute malnutrition (SAM) (HR = 3.3; 95% CI: 2.4–4.9; *p* < 0.001), severe biventricular congenital heart disease (HR = 1.8; 95% CI: 1.2–2.7; *p* = 0.02), severe univentricular congenital heart disease (HR = 11.5; 95% CI: 8.2–19.4; *p* < 0.001), NYHA/modified Ross class III/IV HF (HR = 1.9; 95% CI: 1.1–2.7; *p* = 0.03), PH (HR = 1.4; 95% CI: 1.2–2.3; *p* = 0.02), and an indication for surgical or catheter-based intervention (HR = 1.8; 95% CI: 1.1–4.1; *p* = 0.04).

**Conclusion:**

Acyanotic congenital heart diseases, mainly ventricular septal defect, patent ductus arteriosus, and secundum atrial septal defect, were most common. Over three-quarters of children developed complications, including at admission. Few received the needed surgical or catheter-based interventions. Survival was low, exceeding developed-country mortality. Syndromic features, advanced HF, PH, congenital heart disease complexity, and intervention need predicted mortality, highlighting the significance of early diagnosis and care.

**Supplementary Information:**

The online version contains supplementary material available at 10.1186/s12872-026-06009-y.

## Introduction

Congenital heart disease (CHD) encompasses structural abnormalities of the heart and great vessels present at birth, including defects in venous drainage, septation, alignment, and valvular function [1, 2]. The current birth prevalence of CHD is estimated at 5–11 per 1,000 live births, corresponding to an incidence of approximately 1% (1). Nevertheless, the burden of CHD has not remained static; between 1990 and 2021, the global prevalence of childhood CHD increased by 3.4%, largely due to improvements in diagnostic modalities [3]. Global reports, primarily from hospital- or center-based studies rather than population-level surveillance, suggest that Ventricular septal defect(VSD), Atrial septal defect (ASD), and Patent ductus arteriosus (PDA) are the most common acyanotic CHDs, while Tetralogy of Fallot (TOF) is the most frequent cyanotic defect; however, these data may not fully reflect true population incidence or spectrum [4]. Despite these global estimates, accurately quantifying the true prevalence and patterns of congenital heart disease (CHD) in Africa remains difficult, largely due to limited access to healthcare and consequent underdiagnosis [3, 5, 6]. This limitation is reflected in the scarcity of available evidence, as a meta-analysis including only 10 population-based and 32 hospital-based studies reported population- and hospital prevalences of 5.12 and 12.63 per 1,000 children, respectively [7].

CHD can cause heart failure, arrhythmias, stroke, infections, and death, and in Africa, many children present late with advanced disease [1, 8, 9]. Ethiopian studies report heart failure, pulmonary hypertension, and arrhythmias in adults, and wasting, heart failure, and recurrent infections in children [10, 11] .

Although CHD remained the leading cause of death from congenital birth defects in children under five by 2021, global CHD mortality declined 56.2% since 1990, with the highest rates persisting in low- and low–middle-income countries [3, 12].

Advances in pediatric cardiology and congenital cardiac surgery have greatly improved the diagnosis and treatment of CHD in children, with survival to adulthood increasing most notably among those with the most severe disease; in developed countries, 90% of children diagnosed with Severe complex CHD now survive to age 18 in many settings [13]. In contrast, around 90% of children in Africa with CHD lack access to adequate medical care, with only a few centers providing surgical management. Long waiting lists then result in late presentation, multi-organ complications, and rising CHD mortality of 38.1%, 4.6%, and 40.3% in central, eastern, and western sub-Saharan Africa, respectively [14].

In Ethiopia, data on the pattern of CHD, management, rate of complications and survival, and predictors of mortality among children with CHD remain limited. Therefore, assessing these factors is essential for timely interventions and risk stratification to improve patient outcomes. This study aimed to describe the patterns of CHD and associated complications, show types of management given, quantify survival, and identify predictors of mortality in children under 18, providing data for quality improvement, cross-setting comparisons, epidemiology, and medical education.

## Methodology

### Study area, period, and design

**Fig. 1:**
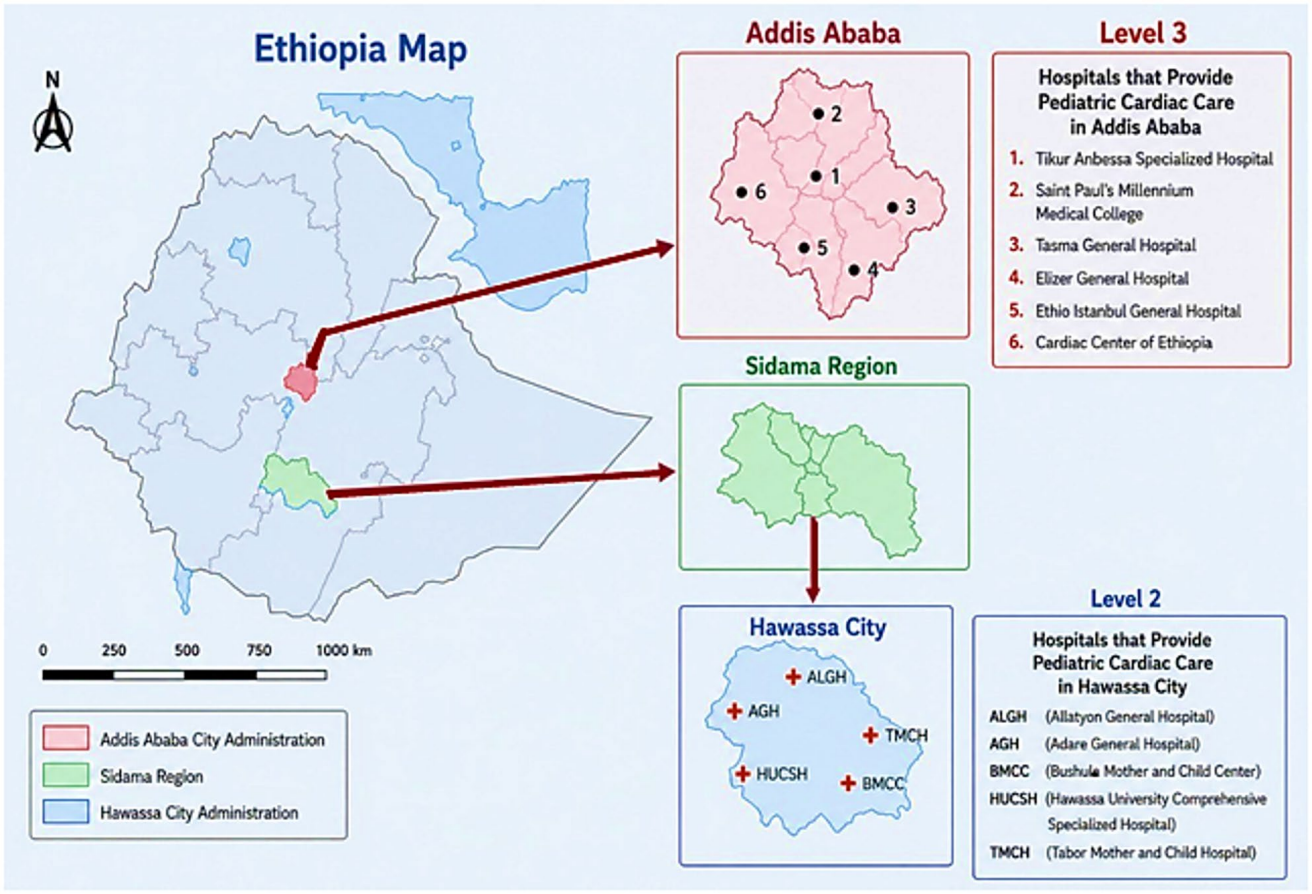
Locations of five hospitals providing pediatric cardiac management in Hawassa, Sidama, Ethiopia, and private, governmental, and charity-based hospitals in Addis Ababa that provide pediatric cardiac surgical and/or catheter-based interventional services. Note: Levels of pediatric and congenital cardiac services were applied as recommended for managing the global burden of pediatric and congenital heart disease (PCHD). The study was conducted in Hawassa across Pediatric and Congenital Cardiac Service Level 2 (PCCSL-2) facilities, with referrals to Pediatric and Congenital Cardiac Service Level 3 (PCCSL-3) centers in Addis Ababa for advanced care

This retrospective multicenter follow-up study was conducted at five hospitals in Hawassa town, Sidama Region, located 273 km south of Addis Ababa, Ethiopia, from April 1 to July 1, 2025. It included patients who attended outpatient and inpatient units between January 1, 2015, and January 1, 2025. These hospitals included Hawassa University Comprehensive Specialized Hospital (HUCSH), Adare General Hospital (AGH), Tabor Mother and Child Hospital (TMCH), Bushulo Mother and Child Center (BMCC), and Allatyon General Hospital (ALGH), which collectively serve over 18 million patients annually from the Sidama Region and neighboring Oromia and Southern regions **(**Fig. 1**)**. Figure 1 also illustrates the hospitals in Addis Ababa to which patients are referred for possible surgical or catheter-based interventions. In line with Ethiopia’s three-level health system, pediatric cardiac services are primarily delivered through outpatient and inpatient pediatric cardiology units, general MCH (Mother-to-Child), and specialized hospitals. In Hawassa, in the aforementioned hospitals, most care is provided by pediatric cardiologists using transthoracic echocardiography (Sonoscape, Samsung, GE-9, and Aloka), chest X-ray, electrocardiography, Holter monitoring, and CT scans.

Globally, integrated tier-based frameworks are recommended for pediatric and congenital heart disease (CHD) care in low- and middle-income countries, but such a framework has not been implemented in Ethiopia [15]. Consequently, most patients are diagnosed only when symptomatic or incidentally, as there is no national CHD screening program. Patients in this cohort were followed at intervals ranging from monthly to every four months, depending on their clinical condition, and were linked primarily to the Cardiac Center of Ethiopia, a charitable center in Addis Ababa, for assessment and potential surgical or catheter-based interventions, while continuing routine follow-up care in Hawassa. In Ethiopia, Level 2 cardiac services, as defined within the cardiac care classification of the Global Initiative for Children’s Surgery (GICS) Cardiac Surgery Working Group, are available in general and specialized hospitals, as well as medium-level clinics across all regions of the country. However, pediatric cardiac care remains highly centralized. The majority of services are concentrated in a single center (the Cardiac Center of Ethiopia), which provides free surgical and catheter-based interventions for approximately three patients per week. In addition, three private centers offer similar services, although these are largely unaffordable for most families. Two government hospitals in Addis Ababa also provide pediatric cardiac surgery; however, their services are limited mainly to procedures such as patent ductus arteriosus (PDA) ligation and pericardiectomy.

### Populations

The source population comprised all children (age < 18 years) with heart disease who had follow-up in the pediatric cardiac outpatient clinics and/or were admitted to the inpatient units of the five hospitals in Hawassa town. The study population included children with CHD who attended outpatient follow-up and/or were admitted to the inpatient units of these hospitals between January 1, 2015, and January 1, 2025, and who met the inclusion criteria.

### Inclusion and exclusion criteria

All children with CHD who had outpatient follow-up and/or were admitted between January 1, 2015, and January 1, 2025, were eligible for inclusion. Children with incomplete medical records on outcome variables, missing echocardiography or ECG data, diagnoses of patent foramen ovale or small patent ductus arteriosus, or non-functional contact phone numbers were excluded.

### Study variables

The dependent variables in this study were the pattern of CHD, the occurrence of complications, survival, and mortality among children with CHD. Independent variables included sociodemographic factors (age at presentation, sex, residence, and region), anthropometric profile (weight, height, mid upper arm circumference-MUAC), clinical characteristics (syndromic association, family history of CHD, presence of heart failure (HF) symptoms at presentation, type and severity of CHD, and presence of complications), and management-related factors, including indication for surgical or catheter-based intervention.

### Data collection techniques, tools, and quality assurance

Data were collected using a structured questionnaire developed after reviewing relevant literature. Patient charts were retrieved using registration numbers from the pediatric cardiac clinic follow-up logbooks. Relevant data were extracted from the medical charts and supplemented by phone interviews for children whose mortality status was not documented, using Kobo-Toolbox. Kobo-Toolbox is an open-source, web-based data collection platform commonly used in humanitarian and health research settings. It allows secure, offline-compatible surveys and efficient management of field-collected data. Senior pediatric residents served as data collectors after receiving a two-day training on the study objectives, data collection procedures, ethical considerations, and confidentiality. The questionnaire was pretested on 5% of the study population at another general hospital in Hawassa, and necessary modifications were made to improve clarity and consistency. During data collection, the principal investigator closely supervised the process and checked the completeness and accuracy daily.

### Operational definitions

CHD was categorized as non-severe, severe biventricular, and severe univentricular to make the classification consistent with a previous study from a developing country [16].

Complications included in this study were heart failure (New York (NYHA)/Modified Ross class III/IV HF, pulmonary hypertension (PH), severe acute malnutrition (SAM), infective endocarditis, recurrent pneumonia, arrhythmia, polycythemia, hyper-cyanotic spells, Eisenmenger syndrome, right and left ventricular dysfunction, major bleeding, stroke, and pulmonary thromboembolism, all of which were identified through a thorough review of the literature on the topic [1, 8, 9].

PH was defined as a peak pulmonary regurgitation (PR) gradient > 20 mmHg or a peak tricuspid regurgitation (TR) velocity > 2.8 m/s on transthoracic echocardiography in the absence of obstruction causing elevated right ventricular pressure. SAM was defined according to WHO criteria: in children under 5 years, weight-for-height/length < − 3 SD or, in those aged 6 months to 5 years, MUAC < 115 mm; in children older than 5 years, it was defined as BMI-for-age < − 3 SD. Stunting was defined as height/length-for-age <- 2 SD, and underweight as weight-for-age <- 2 SD. Anthropometric measurements for children with syndromes were interpreted using growth charts specifically prepared for the respective syndromes. Detailed operational definitions of all other complications are provided in the supplementary file.

Indication for surgical or catheter-based intervention in children with CHD in this study was determined according to the Indian guidelines for indications and timing of intervention for common CHD: revised and updated consensus statement of the Working Group on Management of Congenital Heart Diseases [17]. In this study, syndromes were diagnosed based on characteristic phenotypic features. All-cause mortality was used as the outcome for the mortality analysis in this study.

Survival was defined as the duration from the time of diagnosis of CHD to either death or the end of the study follow-up period.

### Data processing and analysis

Data were initially entered into the Kobo tool, then cleaned, coded, and analyzed using Python (version 3.12). Categorical variables were summarized using frequencies and percentages, while continuous variables (e.g., age at admission) were described using median and interquartile range (IQR) after testing for normality with the Shapiro–Wilk test. The incidence rate of mortality was calculated per 1,000 person-years. Kaplan–Meier survival analysis was used to assess overall mortality, and the log-rank test was applied to compare survival across different CHD severity categories. Cox proportional hazards regression was employed to identify predictors of all-cause mortality, with variables showing p-values < 0.25 in bivariable analysis included in the multivariable model. Adjusted hazard ratios (AHR) with 95% confidence intervals (CI) were used to quantify the strength of associations, and a p-value < 0.05 was considered statistically significant. Data were presented using tables and charts.

## Results

### Sociodemographic and clinical characteristics

Of the 1,291 pediatric cardiac patients reviewed from five hospitals in Hawassa, 40 were excluded due to incomplete records, missing echocardiography or ECG data, or diagnoses of patent foramen ovale, small patent ductus arteriosus, or non-functional contact phone number. The final study population comprised 1,251 patients, 608 (48.6%) from HUCSH, 203 (16.2%) from AGH, 173 (13.8%) from ALGH, 155 (9.2%) from TCH, and 112 (8.9%) from BMCC. The median age of the children was 13.3 months (IQR: 7.2–18.9 months) at presentation. Females accounted for 699 (55.9%) of the study population. The majority of children were from the Sidama Region, 755 (60.4%), followed by the Oromia Region, 333 (26.6%). The majority of children (882, 70.5%) resided in rural areas, 802 (64.1%) were underweight, and 493 (39.4%) were stunted. Syndromic associations were identified in 209 children (16.7%), including Down syndrome in 200 (16.0%), Turner syndrome in 4 (0.3%), Edwards syndrome in 3 (0.2%), and Patau syndrome in 2 (0.2%). Most children were symptomatic at presentation, 1,201 (96.0%) (Table 1).

**Table 1:** Sociodemographic and clinical characteristics of children with congenital heart disease followed at five hospitals in Hawassa, Ethiopia, January 2015–January 2025.

Variables	*N* = 1251
Age at diagnosis in months, median (IQR)	13.3(7.2–18.9)
Sex, n (%)	
Female	699(55.9)
Male	552(44.1)
Residence, n (%)	
Rural	882(70.5)
Urban	369(29.5)
Region, n (%)	
Sidama	755(60.4)
Oromia	333(26.6)
Southern regions	163(13)
Family history of congenital heart disease, n (%)	
No	1216(97.2)
Yes	35(2.8)
Underweight, n (%)	
No	449(35.9)
Yes	802(64.1)
Stunted, n (%)	
No	758(60.6)
Yes	493(39.4)
Syndromic, n (%)	
No	1042(83.4)
Yes	209(16.7)
Symptomatic, n (%)	
No	50(4.0)
Yes	1201(96.0)
Cardiomegaly on CXR, n (%)	
No	250(20.0)
Yes	1001(80.0)

*Abbreviations***: **CXR (Chest X-ray), IQR (Interquartile range)

### Types of congenital heart disease CHD

**Fig. 2:**
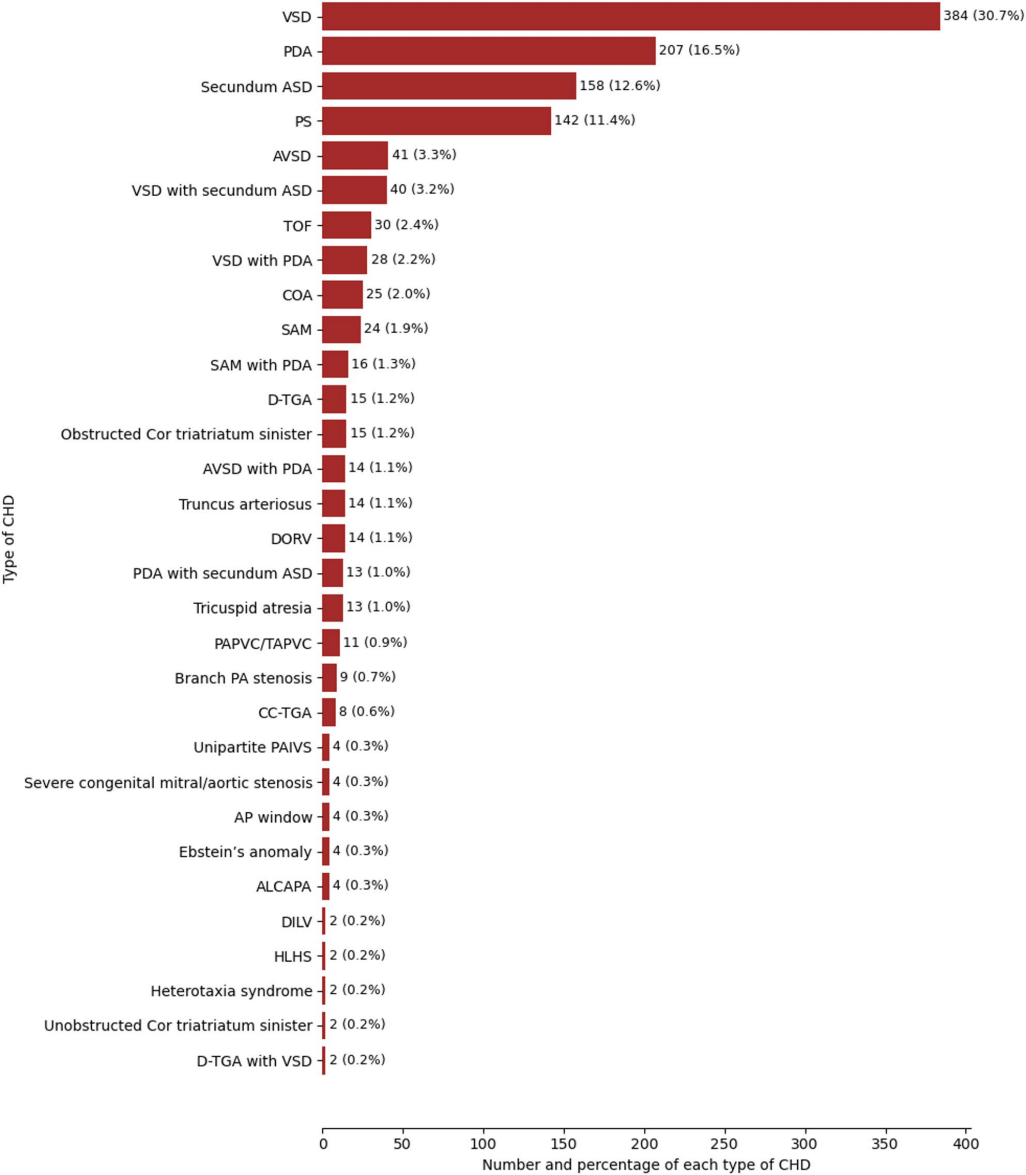
Types of congenital heart disease among children followed at five hospitals in Hawassa, Ethiopia, January 2015–January 2025. Abbreviations: ALCAPA (Anomalous Left Coronary Artery from Pulmonary Artery), AP window (Aortopulmonary Window), AVSD (Atrioventricular Septal Defect), CC-TGA Transposition of the Great Arteries), COA (Coarctation of the Aorta), DILV (Double Inlet Left Ventricle), D-TGA (Dextro-Transposition of the Great Arteries), DORV (Double Outlet Right Ventricle), HLHS (Hypoplastic Left Heart Syndrome), Heterotaxia syndrome (Heterotaxy Syndrome), PAPVC/TAPVC (Partial/Total Anomalous Pulmonary Venous Connection), PDA (Patent Ductus Arteriosus), PS (Pulmonary Stenosis), RV dysfunction (Right Ventricular Dysfunction), SAM (Subaortic membrane), Secundum ASD (Secundum Atrial Septal Defect), TOF (Tetralogy of Fallot), VSD (Ventricular Septal Defect)

Isolated lesions were predominant (1,138; 90.9%). Ventricular septal defect (VSD) was the most frequent isolated lesion (384; 30.7%), followed by patent ductus arteriosus (PDA) (207; 16.5%) and secundum atrial septal defect (ASD) (158; 12.6%). Combined lesions constituted 113 (9.1%). Among combined defects, VSD with secundum ASD was the most common (40; 3.2%), followed by VSD with PDA (28; 2.2%) and atrioventricular septal defect (AVSD) with PDA (14; 1.1%). Acyanotic congenital heart diseases were the predominant diagnoses, accounting for 1,110 cases (88.7%) of the cohort. Cyanotic CHD were less frequent, occurring in 141 cases (11.3%), with Tetralogy of Fallot (TOF) being the leading cyanotic lesion (30; 2.4%), followed by D-transposition of the great arteries (D-TGA) in 15 cases (1.2%), and truncus arteriosus and double outlet right ventricle (DORV), each in 14 cases (1.1%). Based on severity, severe biventricular lesions accounted for 832 cases (66.5%), non-severe lesions for 396 cases (31.7%), and severe univentricular lesions were the least common, occurring in 23 cases (1.8%) (Fig. 2).

### Incidence of complications

**Fig. 3:**
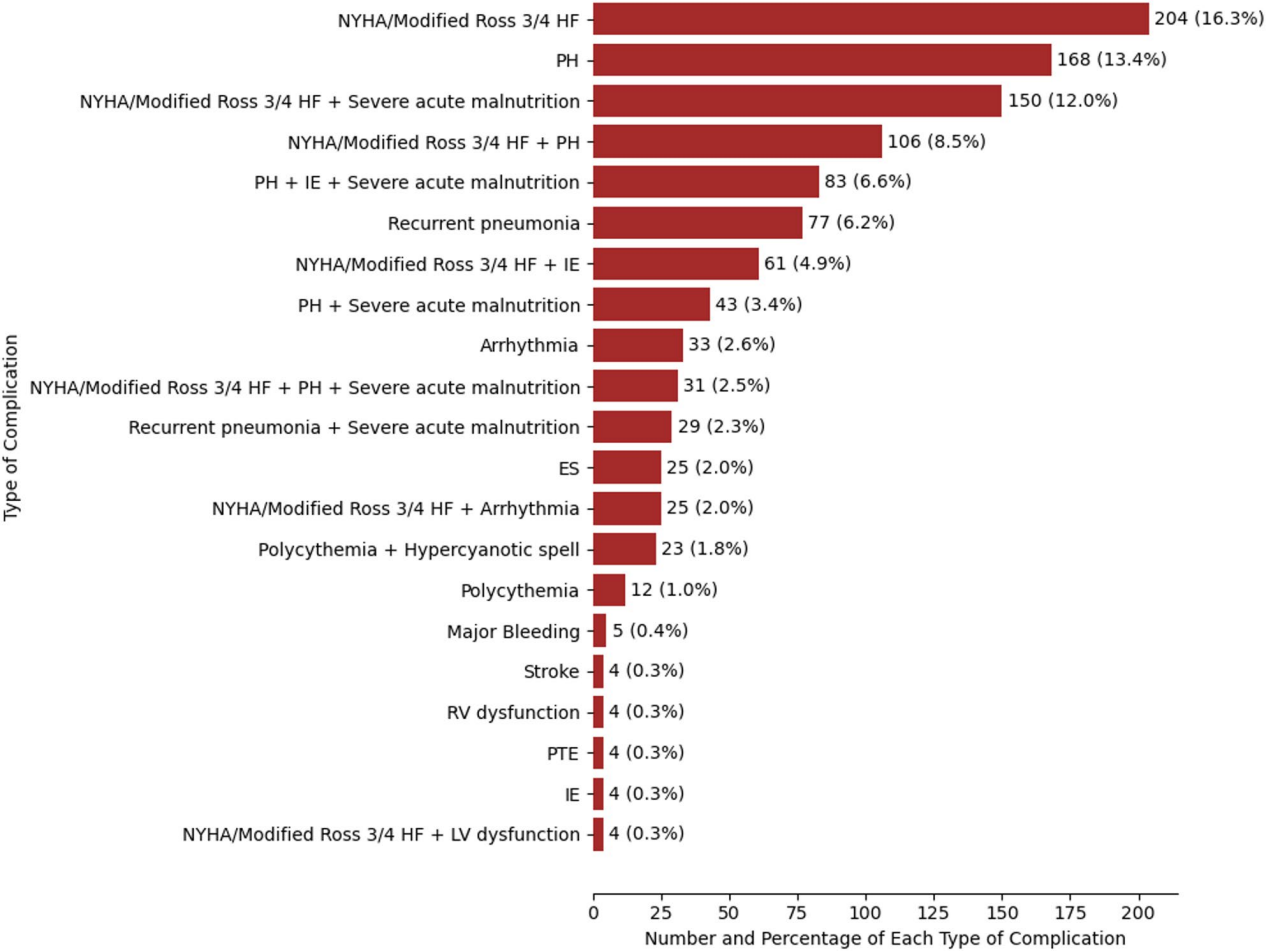
Types of complications among children with congenital heart disease followed at five hospitals in Hawassa, Ethiopia, January 2015–January 2025.Abbreviations: ES (Eisenmenger Syndrome), HF (Heart Failure), IE (Infective Endocarditis), LV Dysfunction (Left Ventricular Dysfunction), NYHA (New York Heart Association Functional Classification), PTE (Pulmonary Thromboembolism), PH (Pulmonary Hypertension), RV dysfunction (Right Ventricular Dysfunction), SAM (Severe Acute Malnutrition)

At presentation, 333 children (31.9%) already had a complication. Over a median follow-up of 5 years (IQR: 4–7), an additional 710 children developed at least one complication, bringing the total to 1,043 children (83.4%) who experienced complications during the follow-up period. Among these, 528 (50.6%) experienced multiple complications, while 515 (49.4%) had a single complication. The most frequent complication was isolated NYHA/Modified Ross class III/IV HF, occurring in 204 children (16.3%), followed by isolated PH in 168 (13.4%), and NYHA/Modified Ross class III/IV HF combined with SAM in 150 (12.0%) (Fig. 3).

### Management and survival

Among 1,251 children with CHD, 1,210 (96.7%) received medical management, 23 (1.8%) had follow-up care only, and 18 (1.4%) underwent surgical or catheter-based intervention at the Cardiac Center of Ethiopia in Addis Ababa (1 catheter-based intervention and 17 surgeries). Among the total cohort, 1,013 children (81.0%) had an indication for surgical or catheter-based intervention according to guideline criteria. However, only 18 children (1.4%) actually underwent the procedure at the Cardiac Center of Ethiopia in Addis Ababa (17 surgeries and 1 catheter-based intervention), meaning the vast majority of indicated children did not receive intervention. During follow-up, survival was 94.4% (17/18) among children who underwent the procedure, compared with 88.3% (879/995) among those who did not. The difference in survival between the groups was not statistically significant (*p* = 0.42). 

**Fig. 4:**
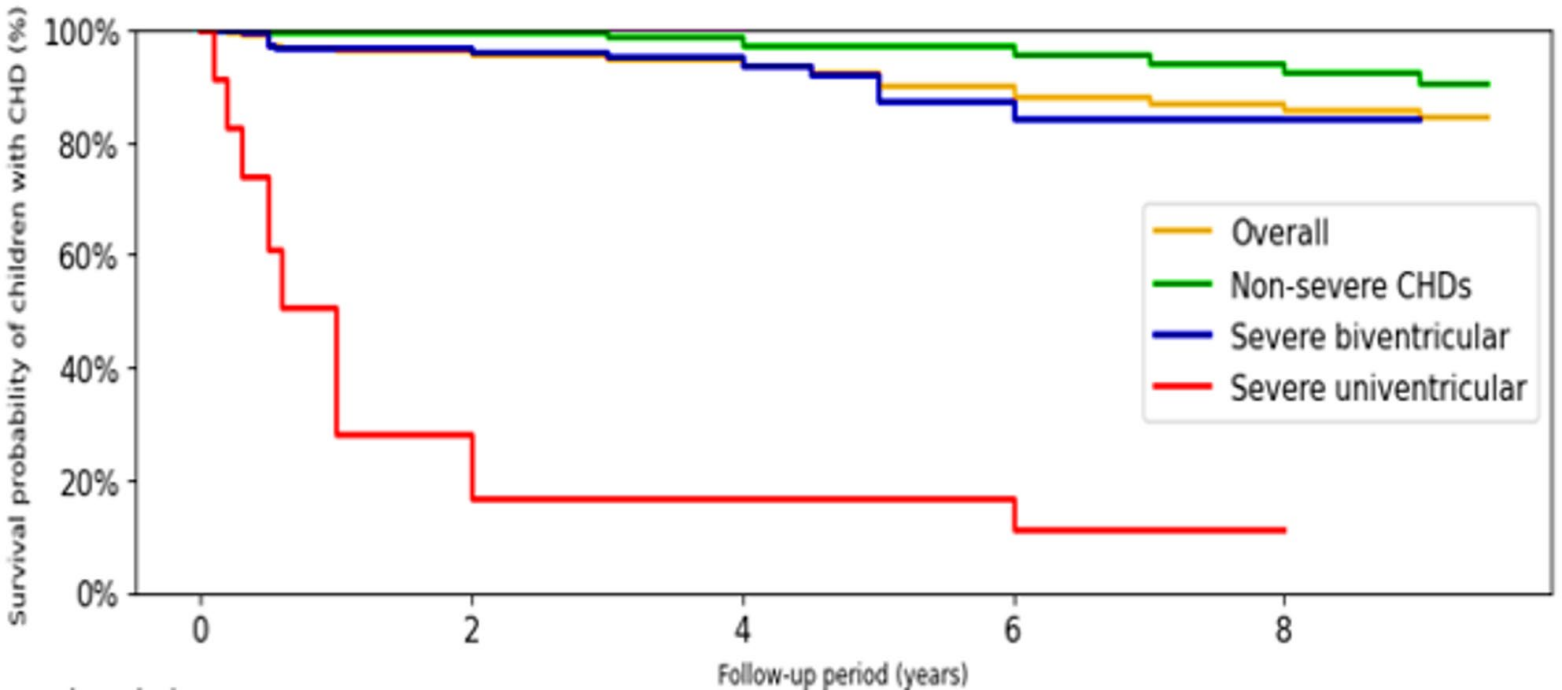
Survival probabilities of children with congenital heart disease by severity category at five hospitals in Hawassa, Ethiopia, January 2015–January 2025.Abbreviation: CHD (Congenital Heart Disease)

The overall survival probabilities for children with CHD were 96.6%, 95.0%, 90.2%, and 84.6% at 1, 3, 5, and 10 years, respectively. Survival varied substantially by CHD severity. At 1 year, survival was 99.5% for non-severe CHDs, 96.9% for severe biventricular CHDs, and 21.7% for severe univentricular CHDs. At 3 years, survival was 98.7%, 95.1%, and 16.9%; at 5 years, 97.2%, 87.5%, and 16.9%; and at 10 years, 90.7%, 84.3%, and 11.3%. Pairwise comparisons showed significantly lower survival for severe univentricular CHDs versus non-severe CHDs and severe biventricular CHDs, as well as for severe biventricular CHDs versus non-severe CHDs (all log-rank p < 0.001) (Fig. 4).

### Incidence rate of mortality

The overall incidence rate of mortality across all types of CHD was 18.9 per 1000 person-years (95% CI 15.9–22.4) over a median follow-up period of 5 years (IQR 4–7). Mortality varied substantially by severity category, being highest in severe univentricular CHDs (18 deaths, 78.3%) at 517.2 per 1000 person-years (95% CI 325.9–821.0), followed by severe biventricular CHDs (86 deaths, 10.3%) at 22.4 per 1000 person-years (95% CI 18.1–27.6), and lowest in non-severe CHDs (26 deaths, 6.6%) at 8.6 per 1000 person-years (95% CI 5.9–12.7). Here, the percentages reflect the proportion of deaths within each severity category, while the incidence rates account for person-time of follow-up. Compared with non-severe CHDs, the incidence rate of mortality was approximately 60 times higher in severe univentricular CHDs (IRR 59.9, 95% CI 32.8–109.2, p < 0.001) and 2.6 times higher in severe biventricular CHDs (IRR 2.6, 95% CI 1.7–4.0, p < 0.001). Of the 130 deaths, 112 (86.2%) occurred in the hospital. The documented causes of in-hospital deaths included respiratory failure (n = 25, 22.3%), cardiogenic shock (n = 23, 20.5%), septic shock (n = 20, 17.9%), and sepsis with multiorgan failure (n = 8, 7.1%); the cause of death was not documented in 36 cases (32.1%). Additionally, 10 deaths (7.7%) occurred en route to the hospital, and 8 deaths (6.2%) were sudden and occurred at home. Of the 112 deaths recorded in the hospital, 78 (69.6%) occurred within the first 48 h of admission. Table 2.

**Table 2:** Predictors of mortality among children with congenital heart disease followed at five hospitals in Hawassa, Ethiopia, January 2015–January 2025

Variables	Total, *n* = 1251	Alive, *n* = 1121	Died, *n* = 130	CHR (95%CI)	AHR (95%CI)	*P* value
Sex, *n* (%)						
Female	699(55.9)	642(57.3)	57(43.8)	1	1	
Male	552(44.1)	479(42.7)	73(56.2)	1.6(1.2–2.3)	0.8(0.5–2.5)	0.32
Residence, n (%)						
Urban	369(29.5)	337(30.1)	32(24.6)	1	1	
Rural	882(70.5)	784(69.9)	98(75.4)	1.3(0.9–1.9)	0.9(0.6–2.1)	0.44
Syndromic, n (%)						
No	1042(83.3)	961(85.7)	81(62.3)	1	1	
Yes	209(16.7)	160(14.3)	49(37.7)	3.0(2.2–4.2)	2.9(2.0-4.3)	0.02
Types of CHD, n (%)						
Acyanotic	1110(88.7)	1023(91.3)	87(66.9)	1	1	
Cyanotic	141(11.3)	98(8.7)	43(33.1)	3.9(2.8–5.4)	0.9(0.5–6.2)	0.07
Severity category of CHD, n (%)						
Non severe	396(31.6)	370(33.0)	26(20.0)	1	1	
Severe biventricular	832(66.5)	746(66.5)	86(66.2)	1.6(1.1–2.4)	1.8(1.2–2.7)	0.02
Severe univentricular	23(1.8)	5(0.4)	18(13.8)	11.9(7.8–18.3)	11.5(8.2–19.4)	< 0.001
NYHA/Ross class III/IV HF, n (%)						
No	670(53.6)	629(56.1)	41(31.5)	1	1	
Yes	581(46.4)	492(43.9)	89(68.5)	2.5(1.8–3.6)	1.9(1.1–2.7)	0.03
PH, n (%)						
No	820(65.6)	763(68.1)	57(43.8)	1	1	
Yes	431(34.4)	358(31.9)	73(56.2)	1.8(1.5–2.1)	1.4(1.2–2.3)	0.02
SAM, n (%)						
No	944(75.5)	882(78.7)	62(47.7)	1	1	
Yes	307(24.5)	239(21.3)	68(52.3)	3.4(2.5–4.6)	3.3(2.4–4.9)	< 0.001
Indication for surgical or catheter-based intervention, n (%)						
No	238(19)	225(20.1)	13(10.0)	1	1	
Yes	1013(81)	896(79.9)	117(90.0)	2.3(1.3–3.9)	1.8(1.1–4.1)	0.04

*Abbreviations*: AHR (Adjusted Hazard ratio), CHR (Crude Hazard ratio), CHD (Congenital Heart Disease), HF (Heart Failure), n (Number), NYHA (New York Heart Association)

### Predictors of mortality

Children with syndromic CHD had an approximately three-fold higher risk of death than those without syndromic features (HR = 2.9; 95% CI: 2.0–4.3; *p* = 0.02). Severe acute malnutrition similarly increased the risk of death by more than three times (HR = 3.3; 95% CI: 2.4–4.9; *p* < 0.001). Mortality rose with increasing disease severity: children with severe biventricular CHD had a 1.8-fold higher risk compared with those with non-severe disease (HR = 1.3; 95% CI: 1.2–2.7; *p* = 0.02), while those with severe univentricular CHD had an over eleven-fold higher hazard of death (HR = 11.5; 95% CI: 8.2–19.4; *p* < 0.001). Among clinical complications, NYHA/ modified Ross class III/IV HF was associated with a 1.9-fold increased mortality risk (HR = 1.9; 95% CI: 1.1–2.7; *p* = 0.03), and PH increased mortality risk by approximately 40% (HR = 1.4; 95% CI: 1.2–2.3; *p* = 0.02). In addition, an indication for surgical or catheter-based intervention was associated with an almost two-fold higher risk of death (HR = 1.8; 95% CI: 1.1–4.1; *p* = 0.04). Although children with cyanotic CHD appeared to have a higher risk of mortality in unadjusted analysis (CHR = 3.9; 95% CI 2.8–5.4), this association was no longer significant after adjusting for disease severity, presence of complications, syndromic features, and nutritional status (AHR = 0.9; 95% CI 0.5–6.2; *p* = 0.07). The wide confidence interval indicates few events in this subgroup, limiting the power to detect an independent effect of cyanotic CHD on mortality.

## Discussion

This study describes the spectrum, complications, and outcomes of CHD in a hospital-based cohort in Ethiopia, where data on this condition are limited. The analysis focuses mainly on unoperated children, providing insights into high-risk groups and informing future resource allocation and intervention strategies.

Consistent with global patterns, acyanotic CHD accounted for the majority of cases in this hospital-based study, with VSD, PDA, and secundum ASD occurring most frequently in descending order of prevalence, and TOF representing the most common cyanotic defect. However, the distribution of specific defects varies by region; for instance, several studies from Asian countries report ASD as the most prevalent acyanotic CHD (4). Such differences likely reflect the lack of population-representative datasets, as hospital-based data may not accurately capture the true incidence and spectrum of CHD across regions.

In this study, a large proportion of children with CHD developed complications (83.4%), with congestive heart failure and PH being the most common. This is similar to findings from other developing countries, including Indonesia (73.4%) and previous studies from Ethiopia, where rates ranged from 70% to 96.3% [11, 18, 19]. The comparable prevalence and patterns of complications may be explained by the predominance of certain CHD types in these studies (VSD, ASD, and PDA among acyanotic defects, and TOF as the most common cyanotic defect), along with delayed diagnosis and late presentation, all of which often result in complications before definitive care [7]. The occurrence of these complications is further increased by limited access to specialized pediatric cardiology and cardiac surgery, inadequate follow-up leading to medication noncompliance, frequent infections that precipitate HF and contribute to PH, and broader health-system constraints common in developing countries [8]. 

Survival in this study was 96.6%, 90.2%, and 84.6% at 1, 5, and 10 years, respectively, which is comparable to findings from Malaysia, where 1-, 5-, and 10-year survival rates were 88%, 85%, and 84% [16]. However, these rates are lower than those reported in developed countries, such as Korea (1-year 98.6%, 5-year 98.3%, and 10-year 98.1%), the USA (10-year survival 90.7%), and Sweden, where 97% of children with CHD survive into adulthood [20–22]. This difference can be explained by delayed diagnosis, limited access to specialized pediatric cardiology and cardiac surgery, and less comprehensive follow-up care in developing countries. 

Children with surgical indications in developing countries often experience prolonged preoperative stress and delayed interventions, regardless of CHD complexity, which contributes to lower survival. In this study, including both simple and complex CHDs, 11.3% of such children died before surgery, a rate comparable to the 11% reported in Malaysia for similar CHD types [16]. 

In this study, although some children presented later than ideal (median 13.3 months), most had lesions suitable for timely surgical intervention. Lessons from Peru and India show that early detection alone is not enough; efficient referral systems, expanded surgical capacity, and population-based screening programs are crucial to improve access, reduce disparities, and decrease mortality [23, 24]. Appropriate combined referral and timely care can substantially reduce preoperative mortality, consistent with international benchmarks such as the International Quality Improvement Collaborative for Congenital Heart Disease (IQIC) 2024 report (< 5% mortality among children requiring surgery) [25]. This underscores the need to strengthen referral systems, improve early diagnosis, and expand access to surgical and catheter-based interventions to improve long-term outcomes.

Multiple studies from both developing and developed countries, similar to ours, have found that the complexity of CHD is associated with lower survival, primarily by increasing mortality [20, 22, 26–29]. Complex CHDs are often more severe, require early and specialized surgical care, and carry a higher risk of complications such as heart failure, arrhythmias, pulmonary hypertension, hypoxemia, stroke, and polycythemia [30]. Children with these CHDs need careful and ongoing long-term follow-up, which can be challenging to provide in many resource-limited settings, further increasing their risk of poor outcomes. 

Consistent with our study, previous research in children, adolescents, and adults has shown that advanced HF and PH are strong predictors of mortality [16, 21, 31–33]. Advanced HF reflects advanced congenital heart disease and impairs cardiac function, while pulmonary hypertension increases right ventricular workload, reduces cardiac output, and worsens tissue oxygenation, together heightening the risk of arrhythmias, multi-organ dysfunction, and death. 

Many children with CHD in this study, as well as in other studies across Africa, were affected by malnutrition, with 26.9% experiencing SAM in this study, 39.4% reported in pooled African data, and in Ethiopia, over half of children with CHD were affected, 54.1% with wasting in one single-center study, and 51.8% with SAM in another [34–36]. SAM was a predictor of mortality in this study, likely due to its effects on immune function, greater susceptibility to infections, reduced tolerance to physiological stress and medical interventions, and overall poor nutritional reserve, all of which worsen outcomes in children with CHD. 

Syndromic associations have been shown to increase the risk of mortality in children with CHD across both developed and developing countries [16, 28, 37, 38]. This likely reflects the added burden these syndromes impose, including additional structural abnormalities, reduced physiological reserve, and increased complexity in medical and surgical management. 

Higher mortality risk among children with CHD requiring surgical or catheter-based interventions in this study likely reflects the combined impact of complex defects, delayed presentation, associated comorbidities, limited access to specialized care, and perioperative risks.

## Conclusion

Acyanotic congenital heart diseases accounted for most cases, with VSD, ASD, and PDA occurring most frequently, while TOF and D-TGA were the predominant cyanotic defects. About one-third of children had complications at presentation, increasing to over three-quarters during follow-up. Although many children had indications for surgical or catheter-based intervention, only a few underwent definitive treatment. Consequently, survival was low, and mortality was higher than that reported in developed settings. Syndromic associations, NYHA class III–IV HF, PH, greater CHD complexity, SAM, and the need for intervention were independent predictors of mortality, demonstrating the importance of early diagnosis and timely access to definitive cardiac care. This study provides the first comprehensive insight into the spectrum, complications, and outcomes of congenital heart disease in Ethiopian children, highlighting gaps in care and opportunities for targeted interventions.

### Limitations of this study

This study has several important limitations. Being retrospective and based on chart reviews, it may be subject to misclassification bias. Syndromic associations were identified solely by physical features, which could lead to under- or over-diagnosis. Pulmonary hypertension was assessed using echocardiography, which may also misclassify some cases. Survival bias is possible, as not all children were followed from birth, and many children with complex CHD may die early in infancy. Despite these limitations, the study provides valuable insights into the patterns of CHD, associated complications, and mortality in children, offering information useful for quality improvement, comparison with other settings, epidemiology, and medical education.

## Supplementary Information

Supplementary Material 1

## Data Availability

All relevant data are available upon reasonable request from the corresponding author.

## Abbreviations

ALCAPA: Anomalous Left Coronary Artery from Pulmonary Artery
AGH: Adare General Hospital
ALGH: Allatyon General Hospital
AP window: Aortopulmonary Window
ASD: Atrial Septal Defect
AVSD: Atrioventricular Septal Defect
BMI: Body Mass Index
BMCH: Bushulo Mother-to-Child Hospital
CHD: Congenital Heart Disease
CHR: Crude Hazard Ratio
COA: Coarctation of the Aorta
CT: Computed Tomography
DILV: Double Inlet Left Ventricle
DORV: Double Outlet Right Ventricle
D-TGA: Dextro-Transposition of the Great Arteries
ECG: Electrocardiography
ES: Eisenmenger Syndrome
HF: Heart Failure
HLHS: Hypoplastic Left Heart Syndrome
HUCSH: Hawassa University Comprehensive Specialized Hospital
IRR: Incidence Rate Ratio
MUAC: Mid-Upper Arm Circumference
NYHA: New York Heart Association Functional Classification
PAPVC/TAPVC: Partial/Total Anomalous Pulmonary Venous Connection
PDA: Patent Ductus Arteriosus
PH: Pulmonary Hypertension
PS: Pulmonary Stenosis
RV dysfunction: Right Ventricular Dysfunction
SAM: Subaortic Membrane
Secundum ASD: Secundum Atrial Septal Defect
TOF: Tetralogy of Fallot
VSD: Ventricular Septal Defect
