# Supplementary material for: Congenital heart disease in children in Hawassa, Ethiopia: a multicenter study on patterns, complications, survival, and mortality predictors
Source: BMC Cardiovasc Disord. 2026 May 26;26:630. doi: 10.1186/s12872-026-06009-y (PMC13393468; doi:10.1186/s12872-026-06009-y)
Supplement: Supplementary file 1 — Supplementary Material 1 [file 12872_2026_6009_MOESM1_ESM.docx]

Complications were defined in this study as follows- infective endocarditis (based on the Modified Duke’s criteria); recurrent pneumonia (≥2 episodes per year or >5 episodes over a lifetime); arrhythmia (tachy- or bradyarrhythmia confirmed by ECG or Holter monitoring); polycythemia (symptomatic if hematocrit >65%, asymptomatic if >70%); hyper-cyanotic spells (sudden episodes of profound cyanosis and hypoxia, often accompanied by dyspnea and irritability, typically occurring in children with cyanotic congenital heart disease); Eisenmenger syndrome (a severe form of pulmonary hypertension characterized by complete reversal of a left-to-right cardiac shunt to a right-to-left shunt, confirmed by transthoracic echocardiography); right ventricular dysfunction (TAPSE <16 mm) and left ventricular dysfunction (ejection fraction <55%); major bleeding (requiring transfusion); stroke (clinically diagnosed and confirmed by CT or MRI); and pulmonary thromboembolism (based on clinical findings supported by echocardiography, ECG, or CT).
